# A First-In-Human Feasibility Study of a New Implantable Tibial Nerve Stimulator for Overactive Bladder Syndrome

**DOI:** 10.1016/j.neurom.2025.01.013

**Published:** 2025-03-01

**Authors:** Parminder S. Sethi, Kenneth M. Peters

**Affiliations:** 1 John Muir Urology, San Ramon, CA, USA;; 2 Department of Urology, Corewell Health William Beaumont University Hospital, Royal Oak, MI, USA; and; 3 Oakland University William Beaumont School of Medicine, Rochester, MI, USA

**Keywords:** Feasibility, overactive bladder, tibial nerve, urgency urinary incontinence

## Abstract

**Objectives:**

This study aimed to evaluate the preliminary feasibility and safety of a new implantable tibial nerve stimulator device in patients with overactive bladder (OAB).

**Materials and Methods:**

Ten patients with OAB in whom conservative treatments had failed participated in the study. All patients received daily stimulation therapy for two weeks and weekly stimulation therapy for 13 weeks, at the lowest electrical output amplitude at which paresthesia or motor response occurred. OAB symptoms (three-day bladder diary), quality-of-life scores (OAB-questionnaire Short Form), and patient global response (GRA) were assessed at each follow-up visit.

**Results:**

Among the patients enrolled (mean age 68 years), seven were OAB-wet and three OAB-dry. After 13 weeks of therapy delivery, a reduction in the number of daily voids during waking hours (8.5 ± 2.5 to 6.3 ± 1.9; *p* = 0.016), incontinence episodes (2.5 ± 1.8 to 0.3 ± 0.5; *p* < 0.001), and the daily voids associated with urgency (7.6 ± 3.1 to 3.0 ± 3.1; *p* < 0.001) was reported. Eight of the ten subjects were classified as responders for ≥one OAB component, with one patient reporting worsened symptoms of urgency (+6%). All ten patients reported global improvement in symptoms on the GRA with a median score of 6. Improvements in OAB severity and health-related quality-of-life scores were noted from pre- to posttreatment across participants (*p* < 0.001). No serious adverse effects were noted.

**Conclusion:**

This first-in-human pilot study allowed for capture of preliminary safety and effectiveness information on a new implantable tibial nerve stimulator device in the population of interest to adequately power a larger, pivotal randomized study.

**Clinical Trial Registration:**

The Clinicaltrials.gov registration number for the study is NCT04115228.

## INTRODUCTION

Overactive bladder (OAB) is defined as a complex syndrome associating urinary urgency, usually accompanied by increased daytime frequency and/or nocturia, with urinary incontinence (OAB-wet) or without (OAB-dry), in the absence of urinary tract infection or other detectable disease.^1^ The severity of urgency and urgency urinary incontinence (UUI) is associated with an important decrease in health-related quality of life (HRQoL), with UUI often reported as the most bothersome symptom.^2^ Epidemiologic studies indicate these symptoms are highly prevalent worldwide (approximately 11%) although true prevalence is likely much higher owing to OAB being underreported and underdiagnosed.^3^

Conservative treatments, including physiotherapy, behavioral therapy, and medications including β-3 agonists, have shown benefit for many patients with OAB. Antimuscarinics currently remain the most used class of medications to treat OAB although adherence can be poor owing to adverse events and lack of efficacy.^4^ In refractory cases, other treatment options include intravesical botulinum toxin (BT), sacral nerve stimulation (SNS), and percutaneous tibial nerve stimulation (PTNS). Even with manifest efficacy in rigorous trials, <15% of patients pursue treatment, and overall utilization of such therapies remains low.^5^ Although PTNS may be a nonsurgical option with minimal significant adverse effects reported, the literature shows contradictory findings in patient preference. The development of new, wireless miniaturized technologies devices allows a minimally invasive simplified neurostimulation procedure without the burden of frequent office visits.^6^ Here, we describe the results from the first-in-human (FIH) feasibility trial using a new, investigational implantable tibial nerve stimulator (ITNS) device in patients with OAB symptoms.

## MATERIALS AND METHODS

### Study Design

This pilot FIH study had a single-arm design and was conducted by Nine Continents Medical, Inc (Livermore, CA). The study provided preliminary data to inform the clinical program design to assess the safety and efficacy of the Intibia^™^ System (Coloplast, Minneapolis, MN), a new ITNS device in development for the treatment of patients with UUI symptoms. The subject eligibility criteria are listed in Table 1. All participants provided written informed consent. The study was conducted in compliance with Title 21 of the Code of Federal Regulations, received ethics approval on May 15, 2019, and was registered on clinicaltrials.gov (NCT04115228).

### Investigational Device

**Figure 1.**
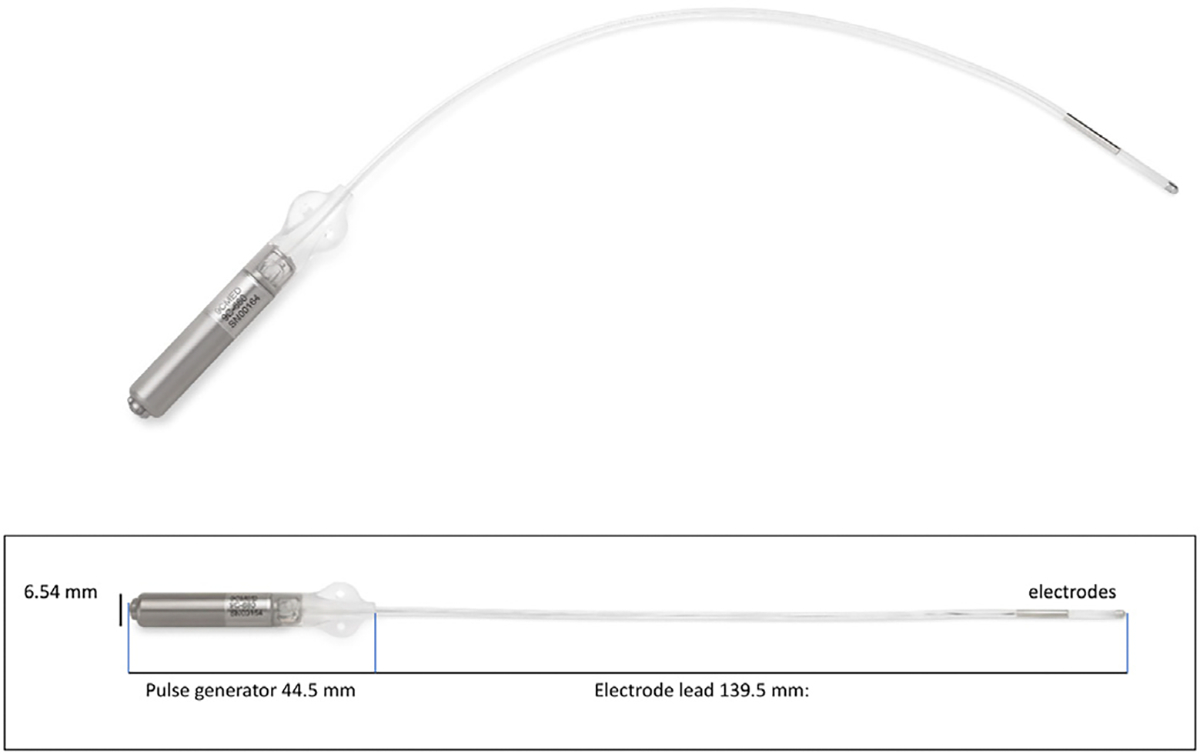
The investigational ITNS. © 2024 Coloplast A/S. All rights reserved. [Color figure can be viewed at www.neuromodulationjournal.org]

The investigational ITNS device used in this trial is an implantable pulse generator (Fig. 1) with an integrated electrode lead permanently implanted through an open procedure in the medial lower leg. The lead is implanted below the fascia at the tibial nerve, and the pulse generator is in the subcutaneous space above the fascia. The ITNS system is magnetic resonance imaging (MRI)-conditional for 1.5T and 3.0T. The pulse generator is powered by a primary nonrechargeable, long-life expectancy lithium cell. It can be interrogated and programmed noninvasively by an external programmer control unit. The pulse generator automatically provides stimulation at a pulse pattern (200 μs) and frequency (20 Hz) preset to match those of tibial nerve stimulation. The pulse amplitude could be set from 0.1V to 10V, allowing tailored treatment to each patient based on motor response and tolerance.

### Patient Screening

At screening, demographics, physical examination, urodynamics, cystoscopy, urinalysis, and pregnancy tests were recorded. All OAB therapies were discontinued. A three-day voiding diary to assess baseline OAB symptoms and quality-of-life scales was completed by the patients. All the patients underwent an evaluation using PTNS, and all had standard sensory and motor findings and tolerated the stimulation.

### Implantation Procedure

To ensure standardized and consistent use of the investigational device, a training program was conducted to educate the investigator and staff, including a cadaver laboratory. The implant comprised an open procedure with local anesthetic and mild sedation, as necessary. A 1.5-to-2 cm incision was made 10 to 12 cm cephalad to the medial malleolus and parallel to the tibia bone. This incision allowed subcutaneous access to create a pulse generator pocket cephalad and implant the lead subfascially. A subcutaneous pocket was then created to house the pulse generator cephalad to the incisional site. Next, a suture was placed transversally through the fascia whereby the implanter then gently tugged upward on the suture, ensuring the suture was through the fascia and not fatty tissue. The implant tool was connected to the system and advanced through the incision and fascia to the target location adjacent to the nerve. Paresthesia or motor response was then verified while stimulating and the electrode lead was then secured. The pulse generator was placed into the subcutaneous pocket and the incision closed.

### Device Programming

After implant, the ITNS device was programmed “off” for a four-week healing period. At the four-week postimplantation follow-up visit, the ITNS device was programmed to threshold output amplitude (defined by the lowest amplitudes at which paresthesia or motor response occurred), and 30 minutes of daily therapy were delivered. After two weeks, the device was reprogrammed to deliver therapy for 30 minutes once per week at a time convenient to the patient to avoid stimulation times that might have interfered with lifestyle activities.

### Evaluation of Symptom Improvement

Alleviation of OAB symptoms (incontinence, frequency, urgency, nocturia) was defined by a ≥50% reduction from baseline; dryness was defined by zero incontinence episodes; and cure was operationalized as a normal frequency defined by ≤seven voids during waking hours. Effectiveness responders were defined by the proportion of subjects indicating ≥50% reduction in ≥one relevant OAB symptom and no worsening of overall relevant symptoms. A three-day voiding diary was used to measure urinary incontinence, frequency, nocturia, urgency, and urgency severity. Quality of life was assessed by the OAB-questionnaire Short Form (OAB-q SF)^7^ and a modified Global Response Assessment (GRA).^8^ OAB-q SF has two subscales: symptom bother (lower score is better) and HRQoL (higher score is better). The modified GRA is a six-point scale ranging from markedly worse (1) to markedly improved (6). The voiding diary and questionnaires were captured at baseline (except for the GRA), and at six and 19 weeks after implant.

Final assessment was undertaken at the 26-week study exit visit. Explant of the ITNS device was planned for any subject with an unplanned dropout or for nonresponse after the 26-week visit. Responders continued per their site standard of care.

### Statistical Analysis

This FIH clinical study was used to capture preliminary safety and effectiveness information on the investigational ITNS device to inform an adequately powered pivotal study in a feasibility sample size of ten patients. All data are presented as means with standard deviation (SD), median with the lowest (minimum) and highest (maximum) values. One-sided *t*-tests were calculated to examine a signal of treatment effect over time within subjects. Statistical significance was defined as *p* ≤ 0.05.

## RESULTS

Sixteen subjects were screened between October 2019 and July 2020. Ten patients (eight women, two men) met the inclusion criteria and provided written informed consent. Mean age was 68 ± 10 years (range 51–85 years). Nine patients reported race as White and one as Asian; 70% of subjects met the study’s inclusion criterion for incontinence, 20% for frequency, 70% for nocturia, and 100% for urgency. Seven patients (70%) were OAB-wet, and three patients (30%) were OAB-dry. Ten individuals were enrolled and implanted with the investigational ITNS device. All patients completed follow-up visits.

### Changes in OAB Symptoms From Voiding Diaries

**Figure 2.**
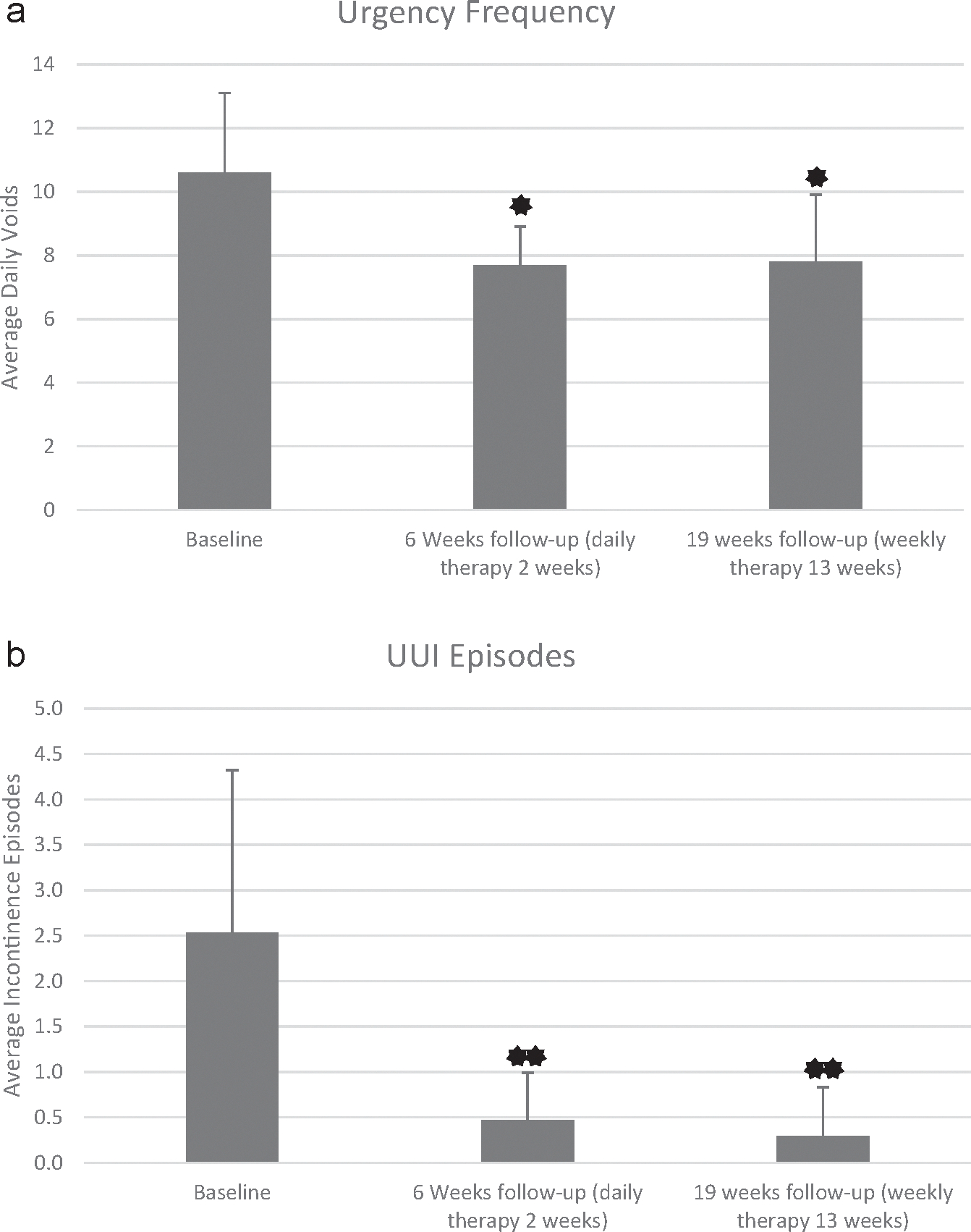
a. Average daily voids per day over time, presented as mean ± SD at baseline, after two weeks of daily therapy and after 13 weeks of weekly therapy. b. Average number of incontinence episodes over time, presented as mean ± SD at baseline, after two weeks of daily therapy and after 13 weeks of weekly therapy. *Indicates change from baseline at a significance level of *p* < 0.05. **Indicates change from baseline at a significance level of *p* < 0.001.

Overall, 80% of patients (eight/ten) were classified as responders (defined as improvements of OAB symptoms [incontinence, frequency, urgency, nocturia] by a ≥50% reduction from baseline; dryness was defined by zero incontinence episodes; cure was operationalized as a normal frequency defined by ≤seven voids during waking hours). Table 2 denotes change from before therapy to after weekly therapy. One patient reported worsened symptoms of urgency and nocturia with UUI, and one patient had no response to treatment. Frequency improved from 8.5 ± 2.5 to 6.3 ± 1.9 episodes per day (*p* = 0.008); total daily voids improved from 10.6 ± 2.5 to 7.8 ± 2.1 episodes per day (Fig. 2a; *p* = 0.002), and the number of leakage episodes decreased from 2.5 ± 1.8 to 0.3 ± 0.5 episodes per day (Fig. 2b; *p* < 0.001).

### Quality of Life and Improvement Questionnaires

All patients completed HRQoL and GRA questionnaires. The patients experienced an increase of the mean HRQoL score from 52.8 ± 29 before therapy to 92.0 ± 12.7 at the end of the weekly therapy (*p* < 0.001) and a decrease of the mean severity score from 58.3 ± 24.5 to 18.3 ± 12.4 (*p* < 0.001) for the same period (Table 2). Improvements on the OAB-q SF correlate with meaningful changes from the patient perspective.^7^

### Safety

No deaths and no serious adverse effects or unanticipated safety concerns were noted during the follow-up. Nine nonserious adverse events occurred in eight subjects. Four patients had incision-site issues including three with redness at the incision site and one with a bacterial culture with positive test results successfully treated with oral antibiotic medication. Two patients had positive urinalysis test results for a urinary tract infection successfully treated with an antibiotic (one of these subjects also experienced incision-site redness). Two subjects successfully underwent previously planned surgeries for unrelated issues. One subject reported that the device stopped working; however, an investigation of the device confirmed normal operation. All subjects exited the study at the 26-week follow-up visit and kept the ITNS implanted.

### Device and Programming Outcomes

**Figure 3.**
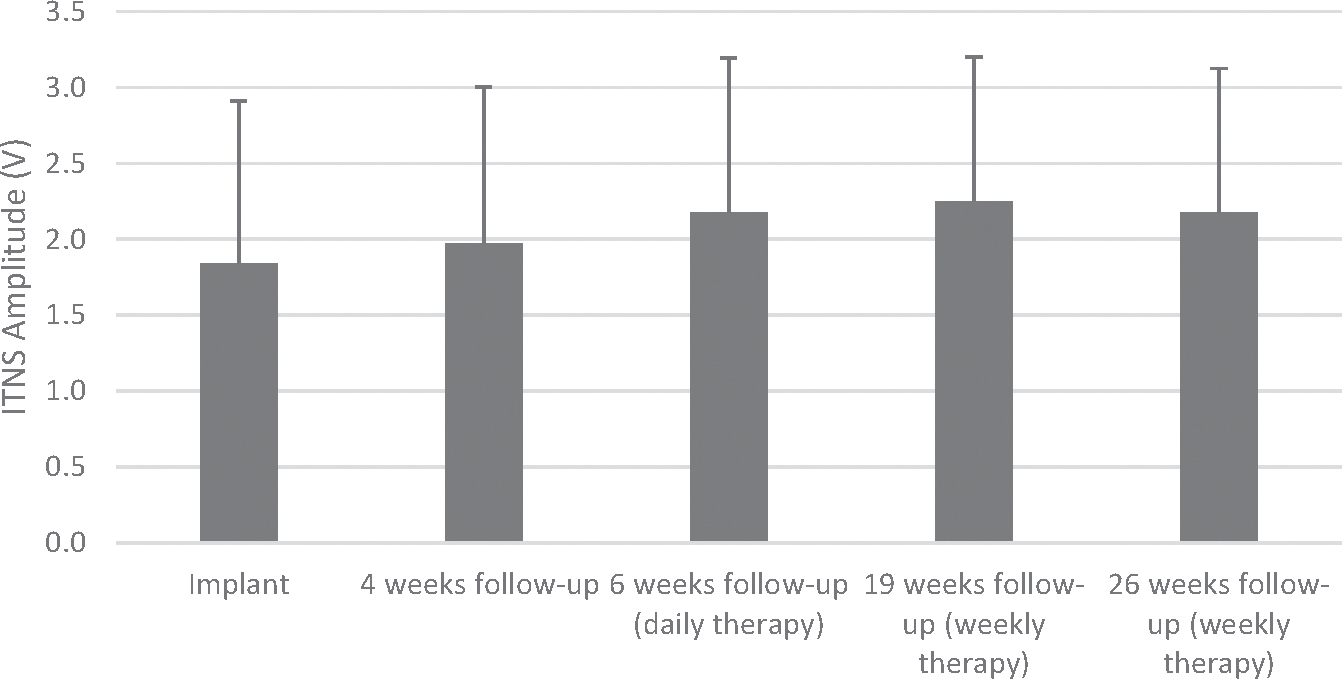
Device amplitude over time, presented as mean ± SD at implant, four weeks, six weeks, 19 weeks, and 26 weeks after implant. ITNS amplitude remained stable over the full 26-week follow-up period.

No significant serious device-related or procedure-related adverse events were reported. No case of migration, pain, or significant increases in voltage output amplitude related to dislodgment was reported over time. Thresholds and programmed amplitudes remained below the maximum ITNS amplitude of 5V (Fig. 3). The average output amplitude across subjects at implant was 1.84V ± 1.07V and was 2.18V ± 0.95V at the 26-week follow-up visit. No device deficiency, electrode migration, or dislodgment was reported.

## DISCUSSION

This study showed short-term improvements in symptoms and quality of life in patients with OAB, with and without UUI, after ITNS therapy. We report decreases in incontinence (89% ± 14%), urinary frequency (90% ± 33%), and urgency (63% ± 37%) from baseline with an average percentage improvement across end points of 69% ± 23% after two weeks of daily and 13 weeks of weekly ITNS therapy. All seven patients who presented with incontinence were deemed responders for UUI, and three of those seven patients were dry, reporting no voids with leakage. Given the deleterious impact UUI symptoms have on patient quality of life, the improvements are encouraging.^2^

The results reported here are similar to those of PTNS, which has shown improvement in urinary symptoms with minimal side effects in patients with refractory OAB. Even if several studies with PTNS indicated efficiency in short-term trials,^9^ maintenance of outcomes over time is lacking. Du et al showed in a real-world study that <40% of patients with PTNS continue to undergo maintenance PTNS therapy after one year,^10^ and as many as 83.5% of patients do not use any third-line therapies.^5^ Te Dorsthorst et al also reported real-life patient experiences of TNS and described reasons for stopping therapy. The combination of loss of efficiency in the long term and low satisfaction rate among patients is predominant.^11^ A core advantage of the new generation of implantable neurostimulators is that limited engagement of the patient is necessary to start and maintain treatment. For device programming, the medical staff adjusts the voltage amplitude between a minimum and maximum range to determine the lowest effective stimulation defined as nonpainful paresthesia or motor responses that the patient could tolerate during 30 minutes of stimulation. The ability to provide sustained daily or weekly stimulation independently from the patient and reduce frequent office visits may improve therapy compliance and efficacy as compared with PTNS.

### Mechanism of Action

**Figure 4.**
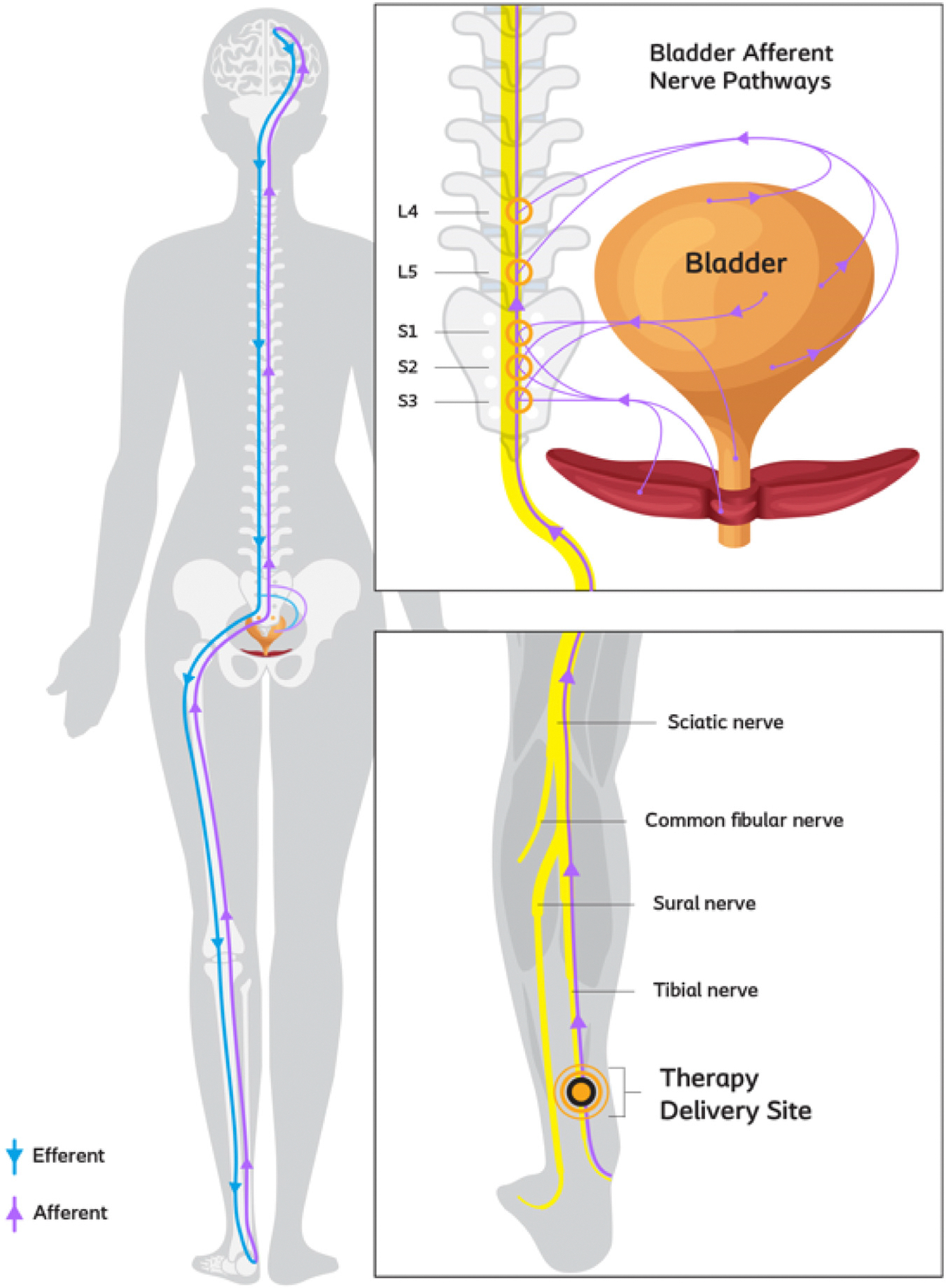
Neuroanatomy and investigational ITNS device placement. The activation of the ITNS device affects afferent signaling of the bladder through interaction of S1, S2, S3, L4, and L5. Providing stimulation at the more distal tibial nerve helps regulate afferent signaling through these nerve roots to the spinal cord and up to the brain. © 2024 Coloplast A/S. All rights reserved.

The results revealed for ITNS are comparable to those found from previous data with other ITNS devices.^12,13^ All the ITNS devices are designed to be implanted in a minimally invasive manner, stimulating the tibial nerve. The main differences are solutions with or without an integrated pulse generator. Although the full ITNS mechanism of action (MoA) has not been fully elucidated, the overall clinical aim is to modulate dysfunctional bladder activity (Fig. 4). Previous research using ITNS has noted significant improvements in bladder function, including delayed first desire to void, first involuntary detrusor contraction, increased maximum cystometric capacity, and reduced urinary retention.^14^

ITNS methods involve the unilateral electrical stimulation of the afferent fibers of the posterior tibial nerve, a mixed nerve containing fibers from the L4-to-S3 nerve roots comprising motor and sensory fibers. The pelvic floor muscle, bladder sphincters, and detrusor muscle are supplied by the same roots. The therapy has been reported to work through spinal cord reflexes and brain involvement through afferent signals (meaning sensory input from the bladder) rather than direct motor modulation of the detrusor or urethral sphincter^12^ (Fig. 4).

### Preclinical Reports

Animal studies have shown that tibial nerve stimulation, at low frequencies (5–30Hz), can induce an inhibitory effect.^15^ A rodent study reported that TNS at 10 Hz produced a maximal inhibitory effect and inhibited bladder contractions immediately after the start of stimulation.^16^ This low level of stimulation may be important given Park et al found that higher frequency at 50 Hz caused an excitatory bladder reflex^12^; therefore, stable stimulation may be important to long-term therapy delivery. McGuire et al were the first to show detrusor activity inhibition in human clinical application with output electrical stimulation parameters ranging from amplitudes of 2V to 8V, frequencies of 2 to 10 Hz, and pulse widths of 5 to 20 milliseconds.^17^ PTNS therapy for OAB currently uses a frequency of 20Hz, similarly to the device reported here.^9^

### Duration of Effect

Tibial nerve stimulation also may have a prolonged post-stimulation inhibitory effect. Research has shown that continuous stimulation for 30 minutes induces both a prolonged inhibition of bladder activity after stimulation and a significantly increased bladder capacity lasting approximately two hours.^18,19^ Human clinical studies have indicated a longer-lasting effect for several days or weeks.^20,21^ Park et al^15^ reported that tibial nerve stimulation has a different MoA to that of sacral nerve or pudendal nerve stimulation, offering a prolonged clinical effect. The stability of the out-amplitude threshold versus time is a strong indication of stability of the position of the ITNS electrode tip regarding the tibial nerve (Figs. 1 and 4).

Finazzi-Agro et al indicated a critical role of the somatosensory pathway and cortical associative structures in tibial nerve stimulation. The authors observed differences in the amplitude of long-latency somatosensory evoked potentials (P80 and P100) after PTNS but not after sham stimulation, which may be indicative of long-term modifications in synaptic efficiency induced by repetitive nerve stimulation and reorganization of the cortical network involved in micturition control.^22^ Numerous techniques such as magnetoencephalography and functional MRI have been used to explore the cerebral areas involved in tibial nerve stimulation. Activation was detected in the postcentral gyrus, posterior parietal cortex, and mesial prefrontal area and bilaterally in the supratemporal region near the Sylvian fissure.^23^ Although a long-latency effect seems to be part of the MoA, additional studies are needed to verify this longitudinally.

### Limitations

Because this was an FIH feasibility study, there are several noteworthy limitations, including the use of a monocentric, non-comparative design. Therefore, conclusions pertaining to relevant clinical benefit cannot be addressed. Furthermore, given the primary etiology of the patient population was diverse, the results cannot be widely extrapolated. A larger, pivotal trial must be operationalized to answer clinical utility over time and generalize the results. Despite these limitations, the initial results were very encouraging. This novel ITNS was shown to reduce urgency frequency and UUI and to improve quality of life primarily in patients who had refractory OAB-wet, offering potentially a new alternative to therapies such as PTNS, SNS, and intravesical BT.

## CONCLUSIONS

This FIH study provided preliminary safety and efficacy data on a new implantable neurostimulator. The preliminary results showed that the ITNS device was safe and efficacious, with notable improvements in most subjective and objective OAB outcomes. A larger, randomized controlled trial is warranted.

## Figures and Tables

**Table 1.** Inclusion and Exclusion Criteria.

Inclusion criteria
Age ≥18y
Documented diagnosis of OAB
Documented failure of behavioral intervention and/or physical therapy
Documented failure of ≥two pharmacotherapies for OAB
Life expectancy of ≥1 y
Capable of tolerating the implantation procedure
Ambulatory and able to use the toilet independently and without difficulty
Able to sense and tolerate posterior tibial nerve stimulation through a transcutaneous preimplantation test
≥One of these criteria based on the 3-d pretherapy bladder diary:
• Average daily voids not awakening from sleep to urinate ≥11
• Average daily voids awakening from sleep to urinate ≥2
• Average daily voids associated with urgency reported as some, severe, or leaked ≥4
• Average daily incontinence episodes ≥1
Exclusion criteria
Predominant stress urinary incontinence
Pelvic organs prolapse with POP-Q ≥grade II for women
Neurologic condition, eg, MS, Parkinson’s disease
Neurogenic bladder (ie, neurogenic lower urinary tract dysfunction)
Abnormalupper urinary tract function
Urinary tract mechanical obstruction due to urethral stricture, due to bladder neck contracture, due to benign prostate hypertrophy in men
Bladder stone or tumor
BMI >40
Chronic pain
Peripheral neuropathy
History of failed neuromodulation for OAB
End-stage renal failure, GFR <35, or dialysis
Untreated diabetes or A1C >7
Implanted pacemaker, defibrillator, or neurostimulator
Condition requiring MRI
Condition requiring diathermy
Metallic implant in planned site of investigational device
Women of child-bearing potential and not willing to practice a medically approved method of birth control during the study
Positive pregnancy test
Women planning to become pregnant
Women having given birth in the last 6 mo
Skin lesions or compromised skin at the implant site
Current urinary tract infection, eg, cystitis, urethritis
Postvoid residual >150cc
Vesico-renal reflux
Gross hematuria

BMI, body mass index; GFR, glomerular filtration rate; MS, multiple sclerosis; POP-Q, Pelvic Organ Prolapse Quantification system.

**Table 2.** Efficacy Results.

From bladder diary (Mean ± SD;median [mini; max])	Before therapy (*n* =10)	After weekly therapy (13 wk) (*n* =10)	*p* Value
Daily voids during waking hours	8.5 ± 2.5 7.8 [5.3; 12.0]	6.3 ± 1.9 6.3 [3.7; 9.7]	0.008
Daily woke up to urinate	2.1 ± 0.8 2.2 [1.0; 3.0]	1.5 ± 0.6 1.5 [0.3; 2.3]	0.005
Total daily voids	10.6 ± 2.5 10.5 [8.0; 15.0]	7.8 ± 2.1 7.3 [5.3; 12.0]	0.002
Daily voids associated with urgency	7.6 ± 3.1 7.0 [4.3; 12.7]	3.0 ± 3.1 2.2 [0.0; 9.0]	<0.001
Daily incontinence episodes	2.5 ± 1.8 2.5 [0.0; 5.0]	0.3 ± 0.5 0.0 [0.0; 1.7]	<0.001
Self-reported severity of urgency:			
Voids with no urgency	0.7 ± 1.2 0.2 [0.0; 3.3]	2.3 ± 2.6 1.5 [0.0; 6.7]	0.016
Voids with mild urgency	2.2 ± 3.2 1.2 [0.0; 10.3]	2.5 ± 2.4 1.3 [0.3; 6.3]	0.386
Voids with some urgency	3.5 ± 2.8 3.3 [0.0; 8.7]	2.2 ± 2.4 1.8 [0.0; 7.7]	0.166
Voids with severe urgency	1.6 ± 1.9 1.2 [0.0; 5.7]	0.5 ± 0.7 0.0 [0.0; 2.0]	0.035
Severity score (OAB-q SF)	58.3 ± 24.5 53.3 [26.7; 96.7]	18.3 ± 12.4 21.7 [0.0; 33.3]	<0.001
HRQoL score (OAB-q SF)	52.8 ± 29.0 54.6 [7.7; 87.7]	92.0 ± 12.7 96.6 [58.3; 100.0]	<0.001
GRA score	–	5.8 ± 0.4 6.0 [5; 6]	N/A

Mean and median voids over the first 72 hours of bladder diary; severity of urgency was scored in five levels: none, mild, some, severe, and leaked.

OAB-q SF scores of patients when finishing their pretherapy, daily, and weekly therapy voiding diaries and patient GRA score after weekly therapeutic periods. Efficacy results were collected through 13 weeks of weekly therapy delivery.

Significance was defined as *p* < 0.05.

max, maximum;mini, minimum; OAB-q SF, overactive bladder questionnaire-short form.
